# Supplementary material for: Effects of Loigolactobacillus coryniformis K8 CECT 5711 on the Immune Response of Elderly Subjects to COVID-19 Vaccination: A Randomized Controlled Trial
Source: Nutrients. 2022 Jan 5;14(1):228. doi: 10.3390/nu14010228 (PMC8747230; doi:10.3390/nu14010228)
Supplement: Supplementary file 1 [file nutrients-14-00228-s001.zip › nutrients-1531773-supplementary.pdf]

Supplementary material and methods

The severity of the SARS-CoV-2 infection was determined according to NIH criteria as:

- Asymptomatic Infection: Individuals who test positive for SARS-CoV-2 using a virologic test (i.e., a nucleic acid amplification test [NAAT] or an antigen test) but who have no symptoms that are consistent with COVID-19.

- Mild Illness: Individuals who have any of the various signs and symptoms of COVID-19 (e.g., fever, cough, sore throat, malaise, headache, muscle pain, nausea, vomiting, diarrhea, loss of taste and smell) but who do not have shortness of breath, dyspnea, or abnormal chest imaging.

- Moderate Illness: Individuals who show evidence of lower respiratory disease during clinical assessment or imaging and who have an oxygen saturation (SpO2) ≥94% on room air at sea level.

- Severe Illness: Individuals who have SpO2 <94% on room air at sea level, a ratio of arterial partial pressure of oxygen to fraction of inspired oxygen (PaO2/FiO2) <300 mm Hg, respiratory frequency >30 breaths/min, or lung infiltrates >50%.

- Critical Illness: Individuals who have respiratory failure, septic shock, and/or multiple organ dysfunction.

Classified as General Business

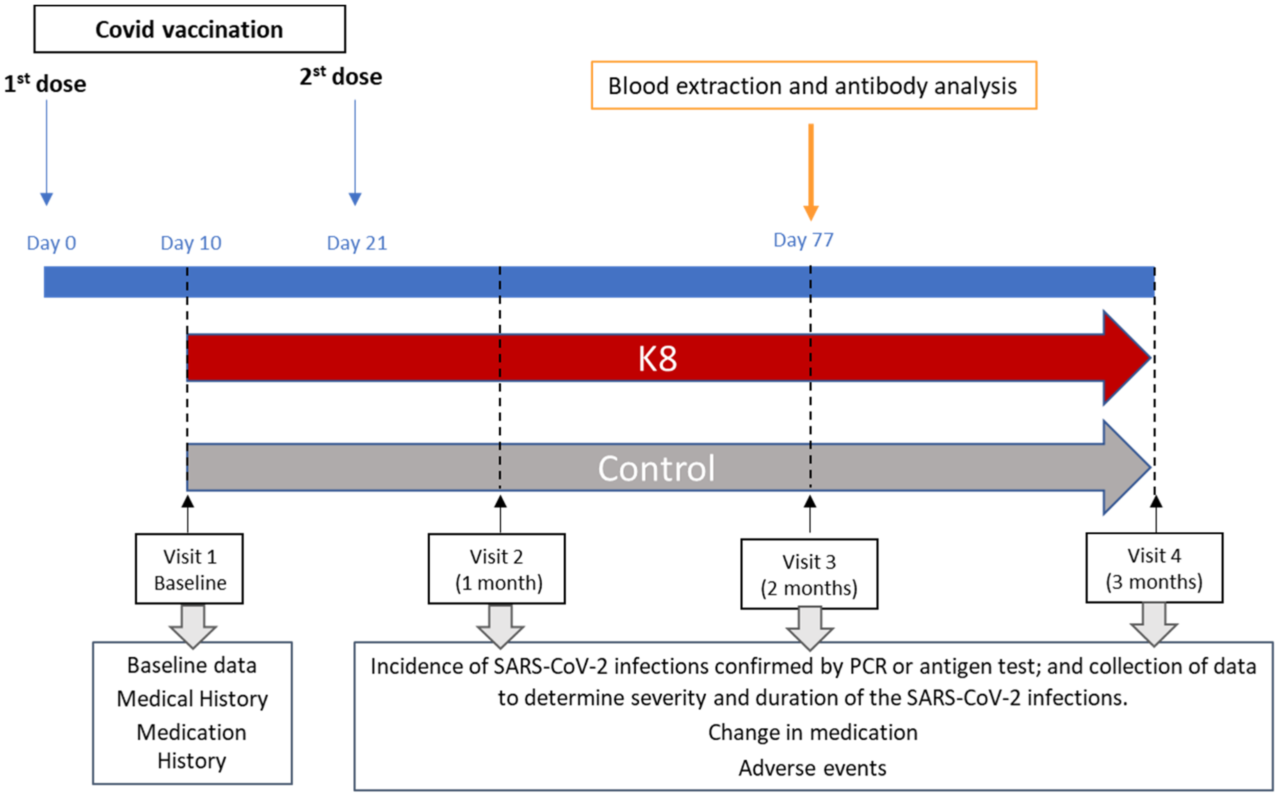

Figure S1. Flow chart of study procedures

Table S1. Baseline medication of the subjects participating in the study.

	All volunteers (n=198)	Control group (n=100)	Probiotic group (n=98)	*P* between-groups
Alzheimer drugs	24 (12.1%)	13 (13.0%)	11 (11.2%)	0.702
Analgesics	101 (51.0%)	49 (49.0%)	52 (53.1%)	0.568
Iron tablets	39 (19.7%)	22 (22.0%)	17 (17.3%)	0.410
Anxiolytics	134 (67.7%)	68 (68.0%)	76 (77.6%)	0.131
Antibiotics	11 (5.5%)	6 (6.0%)	8 (8.2%)	0.553
Antidiabetic drugs	43 (21.7%)	21 (21.0%)	22 (22.4%)	0.805
Antiepileptic drugs	57 (29.1%)	29 (29.0%)	28 (28.6%)	0.947
Antihypertensive drugs	84 (42.2%)	38 (38.0%)	44 (44.9%)	0.357
Parkinson drugs	29 (14.6%)	11 (11.0%)	18 (18.2%)	0.151
Antipsychotic drugs	106 (53.3%)	51 (51.0%)	55 (55.6%)	0.520
Glucocorticoids	11 (5.5%)	4 (4%)	7 (7.1%)	0.343
Diuretics	59 (29.6%)	32 (30.0%)	29 (29.3%)	0.714
Lipids drugs	71 (35.7%)	34 (34.0%)	36 (36.7%)	0.687
Dyspepsia drugs	126 (63.3%)	62 (62%)	64 (64.6%)	0.699
CV drugs	65 (32.7%)	32 (32.0%)	34 (34.7%)	0.688
Vitamins and Food supplements	74 (37.2%)	31 (31.0%)	43 (43.4%)	0.081

Data are presented as n (%). *P* indicates differences between the control group and the probiotic group.

Classified as General Business

Table S2. Summary of statistics for SARS-CoV-2 S1 RBD IgG and IgA antibody response.

	Control group		Probiotic Group		
	**N**	**Geometric mean (95% CI)**	**N**	**Geometric mean (95% CI)**	***P* between-groups ***
**ALL SUBJECTS**					
IgG (U/mL)	97	17,473.93 (730.2-418,141.8)	96	63,676.59 (858.7-509,335.5)	0.552
IgA (U/mL)	97	23,693.66 (121.5-146,923.4)	96	29,326.93 (156.3-184,624.2)	0.422
**COVID-19 PATIENTS**					
IgG (U/mL)	6	50,148.2 (7,480.1-336,202.8)	10	170,054.4 (93,841.8-30,8162.5)	0.038
IgA (U/mL)	6	50,059.2 (9,798.7-255,739.6)	10	73,996.1 (31,961.7-171,312)	0.558
**NO COVID-19 PATIENTS**					
IgG (U/mL)	91	16,300.5 (11,866.5-22,391.2)	86	16,390.4 (11,887.8-22,598.4)	0.579
IgA (U/mL)	91	3,589.7 (2,335.7-5,517)	86	3,959.7 (2,587.9-6,058.6)	0.297
**SUBJECTS <85 years old**					
IgG (U/mL)	43	25,842 (17,781.8-37,555.8)	46	24,531.6 (16,405.5-36,682.7)	0.753
IgA (U/mL)	43	4,389.4 (2,270-8,487.5)	46	3,278.4 (1,826.4-5,884.7)	0.183
**SUBJECTS ≥85 years old**					
IgG (U/mL)	48	10,787.5 (6,716.2-17,326.7)	40	10,308.1 (6,387.9-16,634.1)	0.625
IgA (U/mL)	48	2,997.9 (1,707.6-5,263.3)	40	4,920 (2,645.7-9,149.3)	0.082

Data are presented as geometric mean (95% Confidence Interval) **P* value indicated differences between the probiotic group and the control group in each subgroup analysis (Univariate model adjusted by sex, age and disease index in all subjects; age, sex, disease index and time to Covid-19 symptom resolution in Covid-19 patients; by sex, age, disease index and glucocorticoids in subjects that did not have Covid-19 infections; and by sex, disease index and glucocorticoids in the analysis by age (<85 years old and ≥85 years old)

Classified as General Business

Table S3. Summary of statistics for cytokynes levels

	Control group		Probiotic Group		
	N	Mean (SD)	N	Mean (SD)	*P* between-groups *
**ALL SUBJECTS**					
INF-g (pg/mL)	97	1.04 (3.08)	94	1.36 (3.41)	0.502
TGF-β (pg/mL)	97	12.12 (3.36)	94	11.84 (2.95)	0.575
**COVID-19 PATIENTS**					
INF-g (pg/mL)	6	0.67 (0.74)	9	2.07 (4.14)	0.455
TGF-β (pg/mL)	6	12.77 (4.94)	9	13.48 (2.87)	0.486
**NO COVID-19 PATIENTS**					
INF-g (pg/mL)	91	1.06 (3.18)	85	1.28 (3.34)	0.694
TGF-β (pg/mL)	91	12.08 (3.27)	85	11.66 (2.98)	0.430
**SUBJECTS <85 years old**					
INF-g (pg/mL)	43	0.99 (2.82)	46	1.69 (4.37)	0.618
TGF-β (pg/mL)	43	11.89 (3.00)	46	11.23 (2.48)	0.280
**SUBJECTS ≥85 years old**					
INF-g (pg/mL)	48	1.12 (3.50)	40	0.80 (1.31)	0.988
TGF-β (pg/mL)	48	12.24 (3.52)	40	12.18 (3.34)	0.822

Data are presented as mean (SD). * *P* value indicated differences between the probiotic group and the control group in each subgroup analysis (Univariate model adjusted by sex, age and disease index in all subjects; age, sex, disease index and time to COVID-19 symptom resolution in Covid-19 patients; by sex, age, disease index and glucocorticoids in subjects that did not have Covid-19 infections; and by sex, disease index and glucocorticoids in the analysis by age (<85 years old and ≥85 years old)

Classified as General Business
